# Parental Acceptability of COVID-19 Vaccination for Children Under the Age of 18 Years: Cross-Sectional Online Survey

**DOI:** 10.2196/24827

**Published:** 2020-12-30

**Authors:** Ke Chun Zhang, Yuan Fang, He Cao, Hongbiao Chen, Tian Hu, Ya Qi Chen, Xiaofeng Zhou, Zixin Wang

**Affiliations:** 1 Longhua District Center for Disease Control and Prevention Shenzhen China; 2 Department of Early Childhood Education The Education University of Hong Kong Hong Kong China; 3 JC School of Public Health and Primary Care Faculty of Medicine The Chinese University of Hong Kong Hong Kong China

**Keywords:** parental acceptability, COVID-19 vaccination, children under the age of 18 years, theory of planned behavior, social media influence, China

## Abstract

**Background:**

It is expected that COVID-19 vaccines will become available in China by the end of 2020. Vaccinating children against COVID-19 would contribute to the control of the pandemic and the recovery of the global economy. For children under the age of 18 years, parents are usually the decision makers regarding their children’s vaccination.

**Objective:**

The goal of this study was to investigate parental acceptability of free COVID-19 vaccination for children under the age of 18 years in China.

**Methods:**

This is a secondary analysis of a cross-sectional, closed online survey among 2053 factory workers in Shenzhen, China, implemented from September 1 to 7, 2020. Participants of the online survey were full-time employees aged 18 years or over who had resumed work in factories in Shenzhen. Factory workers in Shenzhen are required to receive physical examinations once a year. Eligible workers attending six designated physical examination sites were invited to complete an online survey. This study was based on a subsample of those who had at least one child under the age of 18 years (N=1052). After being briefed that COVID-19 vaccines developed by China are likely to be available by the end of 2020, participants were asked about their likelihood of having their children under the age of 18 years take up free COVID-19 vaccination provided by the government, if it existed. Multivariate logistic regression models were fitted to examine the associations of perceptions related to COVID-19 vaccination based on the theory of planned behavior (TPB) and exposure to information related to COVID-19 through social media with parental acceptability, after controlling for significant background characteristics.

**Results:**

The prevalence of parents’ acceptability of COVID-19 vaccination for their children was 72.6% (764/1052). After adjusting for significant background characteristics, positive attitudes toward COVID-19 vaccination (adjusted odds ratio [AOR] 1.70, 95% CI 1.50-1.91), the perception that a family member would support them in having their children take up COVID-19 vaccination (ie, perceived subjective norm) (AOR 4.18, 95% CI 3.21-5.43), and perceived behavioral control to have the children take up COVID-19 vaccination (AOR 1.84, 95% CI 1.49-2.26) were associated with higher parental acceptability of COVID-19 vaccination. Regarding social media influence, higher exposure to positive information related to COVID-19 vaccination was associated with higher parental acceptability of COVID-19 vaccination (AOR 1.35, 95% CI 1.17-1.56). Higher exposure to negative information related to COVID-19 vaccination was negatively associated with the dependent variable (AOR 0.85, 95% CI 0.74-0.99).

**Conclusions:**

Parents’ acceptability of COVID-19 vaccination for their children under 18 years of age was high in China. The TPB is a useful framework to guide the development of future campaigns promoting COVID-19 vaccination targeting parents. Transparency in communicating about the vaccine development process and vaccine safety testing is important. Public health authorities should also address misinformation in a timely manner.

## Introduction

Globally, the COVID-19 pandemic remains out of control [1]. The existing measures to control COVID-19 are detrimental to the global economy [2] and result in significant impairment in physical and psychological well-being [3]. There is a strong need for an effective vaccine to keep COVID-19 under control. Development of COVID-19 vaccines is underway. According to the World Health Organization, as of September 3, 2020, there were 34 and 142 candidate vaccines in clinical and preclinical evaluation, respectively; four Chinese candidate vaccines had entered Phase III clinical trials [4]. According to an official press release on September 15, 2020, safety of these Chinese candidate vaccines was established [5]. The National Health Commission of the People’s Republic of China authorized the emergency use of COVID-19 vaccines on July 22, 2020 [6]. COVID-19 vaccines were provided to workers, students, and diplomatic personnel who needed to travel abroad, as well as to health care workers and personnel working for pandemic and border control [5]. It is expected that at least one COVID-19 vaccine will become available in China by the end of 2020 [5,7,8].

The United States National Academies of Sciences, Engineering, and Medicine has proposed a five-phase plan to fairly allocate a COVID-19 vaccine. Health care workers, older adults, and other people with underlying conditions that put them at high risk of severe COVID-19 diseases or death are priority groups to receive the vaccine, followed by essential workers, children, and young adults [9]. Without COVID-19 vaccination, children will likely serve as a reservoir, which would undermine efforts to end the pandemic [10]. Moreover, it is difficult to recover the economy completely before all children can safely return to schools and parents can resume full-time work [10]. According to the aforementioned official press release, health care workers, older adults, and children are considered priority groups to receive COVID-19 vaccination in China [5].

Mathematic modeling suggested that if the COVID-19 vaccine efficacy was 80%, the coverage would have to achieve at least 75% to extinguish the ongoing pandemic [11]. Therefore, a timely understanding of community responses to the forthcoming COVID-19 vaccines are important for policy making and service planning. For children under the age of 18 years, parents are usually the decision makers regarding their children’s vaccination. Hence, it is important to understand parents’ acceptability of their children’s COVID-19 vaccination and related barriers and facilitators. To our knowledge, at least one study investigated parents’ acceptability of COVID-19 vaccination for their children [12]. This was an online survey conducted in the United Kingdom, which showed that 48.2% of parents or guardians would definitely accept COVID-19 vaccination for their children aged 18 months or under [12]. Belief that COVID-19 vaccination could protect their children and other family members and belief that it would facilitate their return to normal life were major reasons for parents’ acceptance of COVID-19 vaccination for their children [12]. Their concerns were around COVID-19 vaccine safety and effectiveness [12]. These factors were considered by this study. We applied the theory of planned behavior (TPB) as the theoretical framework in this study [13]. The TPB postulates that behavioral intention to adopt a health-related behavior (eg, having children take up COVID-19 vaccination) is a strong predictor of actual behavior. In order to form such an intention, one would evaluate the pros and cons of the behavior (ie, positive and negative attitudes), consider whether their significant others would support such behavior (ie, perceived subjective norm), and appraise how much control one has over the behavior (ie, perceived behavioral control) [13]. In recent studies, the TPB has been used successfully to explain vaccination behaviors [14-16].

Previous studies showed that people are actively seeking information about COVID-19 vaccination on social media platforms [17]. Exposure to COVID-19–specific information on social media influenced Chinese factory workers’ adoption of personal preventive measures (eg, face mask wearing, hand hygiene, and physical distancing) [18]. Moreover, different content related to COVID-19 may have varying effects on health outcomes [18]. A previous study suggested that TPB-related constructs and subsequent behavior may be shaped by the use of social media [19]. In this study, we investigated the associations between exposure to different content related to COVID-19 vaccination on social media and parental acceptability of having their children vaccinated.

To our knowledge, there have been no studies investigating parental acceptability of COVID-19 vaccination for their children in China. This study investigated parental acceptability of free COVID-19 vaccination for children under the age of 18 years among parents in Shenzhen, China. We examined the effects of factors on their acceptability, including background characteristics, perceptions related to COVID-19 vaccination based on the TPB, and exposure to information related to COVID-19 vaccination through social media.

## Methods

### Study Design

This is a secondary analysis of a cross-sectional, closed online survey among 2053 factory workers in Shenzhen, China, implemented from September 1 to 7, 2020.

### Participants and Data Collection

Participants of the closed online survey were full-time employees of factories in Shenzhen aged 18 years of age or older. This study was conducted in Longhua District of Shenzhen. The majority of factories in Shenzhen are located in Longhua District; there were over 2000 factories and one million factory workers in 2018 in this district. In Shenzhen, factory workers are required to receive physical examinations at designated sites once a year. All six designated sites providing physical examination services to factory workers in Longhua District, including three public hospitals, two private hospitals, and the district Centre for Disease Control and Prevention (CDC), were chosen as our study sites for recruitment.

To avoid selection bias, the fieldworkers approached all adults attending these sites for physical examinations during the study period. They briefed prospective participants about the study details, confirmed their eligibility, and invited them to join the study. Participants were guaranteed that participation was voluntary, refusal would have no effect on them, the survey would not collect personal contact information or identification, and data would be kept strictly confidential and would only be used for research purposes. Verbal consent was obtained instead of written consent to allow participants to maintain anonymity. We developed an online questionnaire using Questionnaire Star (Changsha Ranxing Information Technology Co), a commonly used online survey platform in China. Quick response (QR) codes were generated to access the online questionnaire. Prospective participants were asked to scan the QR code on-site to complete the survey. Each mobile device was allowed to access the online questionnaire once to avoid duplicate responses. The participants were asked not to disseminate the QR codes that were used to access the survey to other people. The survey had 66 items, approximately 15 items per page for four pages, which took about 15 minutes to complete. The online survey platform performed completeness checks before each questionnaire was submitted. Participants were able to review and change their responses through a *back* button. In case there was more than one child under the age of 18 years within their household, participants referred to the one whose birthday was closest to the survey date when answering questions [20]. Upon completion of the survey, an electronic coupon of ¥10 (US $1.30) was sent to participants. All data were stored in the server of the online survey platform and were protected by a password. Only the corresponding author had the access to the database.

Out of 2653 eligible factory workers being approached, which included between 60 and 1200 across the study sites, 2053 completed the online survey, which amounted to between 40 and 968 across the study sites. The overall response rate was 77.4% (2053/2653); this ranged from 66.7% to 80.7% at different sites. Main reasons for nonresponse were lack of time and other logistic reasons. This study was based on 1052 participants who had at least one child under the age of 18 years. Ethics approval was obtained from Longhua District CDC (reference No. 2020001).

### Measures

#### Development of the Questionnaire

A panel consisting of one CDC staff member, two public health researchers, a health psychologist, a senior factory manager, and a factory worker was formed to develop the questionnaire used in this study. The questionnaire was pilot-tested among 10 factory workers to assess clarity and readability. These 10 workers did not participate in the actual survey. Based on participants’ comments, the panel revised and finalized the questionnaire.

#### Background Characteristics

Participants were asked to report on sociodemographics (ie, age, gender, relationship status, education level, monthly personal income, etc), age of their children, parental history of seasonal influenza vaccination, and whether they had a family member with a history of COVID-19. In addition, participants were asked to report on their frequency of wearing face masks when having close contacts with others in the workplace and in other public settings (ie, public spaces and transportation) in the past month; response categories included *every time*, *often*, *sometimes*, and *never*. Participants also reported on the frequency of sanitizing their hands using soaps, liquid soaps, and alcohol-based hand rubs after returning from public spaces, touching public installations, and touching equipment, and whether they avoided social and meal gatherings with people who do not live together as well as crowed places in the past month.

#### Parental Acceptability of Free COVID-19 Vaccination for Children Under the Age of 18 Years

Participants were briefed with the following statement: “COVID-19 vaccines developed by China are likely to become available by the end of 2020.” They were then asked about the likelihood of having their children under the age of 18 years take up free COVID-19 vaccination provided by the government, if it existed; response categories included the following: 1 (very unlikely), 2 (unlikely), 3 (neutral), 4 (likely), and 5 (very likely). Parental acceptability of COVID-19 vaccination was defined as the responses *likely* or *very likely*. Such definition has been commonly used in previous studies [20,21].

#### Perceptions Related to COVID-19 Vaccination Based on the TPB

Two scales were constructed to assess perceptions related to COVID-19 vaccination based on the TPB. These scales were the 3-item Positive Attitude Scale (eg, “COVID-19 vaccination is highly effective in protecting your child from COVID-19”) and the 4-item Negative Attitude Scale (eg, “Your child will have severe side effects after receiving COVID-19 vaccination”); response categories were as follows: 1 (disagree), 2 (neutral), and 3 (agree). The Cronbach α values of these two scales were .71 and .64, respectively; single factors were identified by exploratory factor analysis, explaining 64.0% and 56.6% of the total variance, respectively. Perceived subjective norm (ie, “Your family member will support you in having your child take up COVID-19 vaccination”) and perceived behavioral control (ie, “Having your child receive COVID-19 vaccination is easy for you if you want them to”) were measured by two single items; the response categories were as follows: 1 (disagree), 2 (neutral), and 3 (agree).

#### Influence of Social Media

Participants were asked to report on the frequency of their exposure to information related to COVID-19 vaccination on social media (ie, WeChat, WeChat Moments, Weibo, TikTok, etc) in the past month; response categories were as follows: 1 (almost none), 2 (seldom), 3 (sometimes), and 4 (always). Such information included (1) positive information related to COVID-19 vaccination (eg, new vaccines entering clinical trials), (2) negative information related to COVID-19 vaccination (eg, concerns about vaccine efficacy, supply, and side effects and about the receipt of vaccines causing COVID-19), (3) testimonials given by participants of the COVID-19 clinical trials, and (4) negative information about vaccine incidents in China (eg, selling problematic vaccines and severe side effects).

### Statistical Analysis

Parental acceptability of COVID-19 vaccination was used as the dependent variable. A univariate logistic regression model first assessed the significance of the association between each of the background characteristics and the dependent variable. Background characteristics with *P*<.05 in the univariate analysis were adjusted in a multivariate logistic regression model. Principal component analysis with varimax rotation was used to perform explanatory factor analysis. SPSS Statistics for Windows, version 26.0 (IBM Corp), was used for data analysis, with *P*<.05 considered statistically significant.

## Results

### Background Characteristics

Over half of the participants were 40 years old or younger (824/1052, 78.3%), were female (658/1052, 62.5%), were married (1005/1052, 95.5%), did not receive tertiary education (785/1052, 74.6%), had a monthly income level lower than ¥5000 (US $714) (703/1052, 66.8%), and were frontline workers (701/1052, 66.6%). Among the parents, 20.0% (210/1052) had received seasonal influenza vaccination in the past and 0.2% (2/1052) had at least one family member with a history of COVID-19. About half of their children were 0 to 6 years of age (490/1052, 46.6%).

In the past month, 72.1% (759/1052) and 83.1% (874/1052) of participants reported wearing a face mask every time they had close contact with other people in the workplace and in other public settings, respectively. Fewer participants self-reported sanitizing their hands (606/1052, 57.6%), avoiding social and meal gatherings (622/1052, 59.1%), and avoiding crowed places (697/1052, 66.3%) (see Table 1).

**Table 1:** Background characteristics of the parents.

Characteristics			Value (N=1052), n (%)
**Sociodemographics**			
	**Age of the parent (years)**		
		18-30	238 (22.6)
		31-40	586 (55.7)
		>40	228 (21.7)
	**Gender**		
		Male	394 (37.5)
		Female	658 (62.5)
	**Relationships status**		
		Married	1005 (95.5)
		Currently single or divorced	43 (4.1)
		Having a stable boyfriend or girlfriend	4 (0.4)
	**Highest education level attained**		
		Junior high school or below	448 (42.6)
		Senior high school or equivalent	337 (32.0)
		College or university or above	267 (25.4)
	**Monthly personal income (¥)**		
		<3000	238 (22.6)
		3000-4999	465 (44.2)
		5000-6999	185 (17.6)
		7000-9999	90 (8.6)
		≥10,000	74 (7.0)
	**Type of work**		
		Frontline worker	701 (66.6)
		Management staff	351 (33.4)
	**History of seasonal influenza vaccination**		
		No	842 (80.0)
		Yes	210 (20.0)
	**Having at least one family member with a history of COVID-19**		
		No	1050 (99.8)
		Yes	2 (0.2)
	**Age of the child (years)**		
		0-3	314 (29.8)
		4-6	176 (16.7)
		7-12	373 (35.5)
		13-17	189 (18.0)
**Personal COVID-19 preventive measures in the past month**			
	**Frequency of face mask wearing in public places and on transportation other than the workplace**		
		Every time	874 (83.1)
		Often	139 (13.2)
		Sometimes	36 (3.4)
		Never	3 (0.3)
	**Frequency of face mask wearing when you have had close contact with other people in the workplace**		
		Every time	759 (72.1)
		Often	204 (19.4)
		Sometimes	82 (7.8)
		Never	7 (0.7)
	**Self-reported sanitizing of hands, using soaps, liquid soaps, or alcohol-based sanitizer, after returning from public spaces or touching public installations**		
		Every time	606 (57.6)
		Often	277 (26.3)
		Sometimes	156 (14.8)
		Never	13 (1.2)
	**Self-reported avoiding of social and meal gatherings with other people who do not live together**		
		No	430 (40.9)
		Yes	622 (59.1)
	**Self-reported avoiding of crowed places**		
		No	355 (33.7)
		Yes	697 (66.3)

### Parental Acceptability, Perceptions, and Influences of Social Media Related to COVID-19 Vaccination

Among the parents, the prevalence of parental acceptability of free COVID-19 vaccination was 72.6% (764/1052) (see Table 2). Individual item responses and mean (SD) values of the scales related to parental perceptions of COVID-19 vaccination are presented in Table 2. Among the participants, 69.3% (729/1052) were sometimes or always exposed to positive information related to COVID-19 vaccination in the past month. Among the participants, fewer were sometimes or always exposed to negative information related to COVID-19 vaccination (442/1052, 42.0%) or vaccine incidents in China (298/1052, 28.3%), or were sometimes or always exposed to testimonials given by participants of COVID-19 vaccination clinical trials (283/1052, 26.9%) (see Table 2).

**Table 2:** Perceptions related to COVID-19 vaccination.

Acceptability and perceptions				Value (N=1052), n (%) or mean (SD)
**Parents’ acceptability of COVID-19 vaccination for their child under the age of 18 years: likelihood of having the child take up free COVID-19 vaccination, n (%)**				
	Very unlikely		19 (1.8)	
	Unlikely		45 (4.3)	
	Neutral		224 (21.3)	
	Likely		361 (34.3)	
	Very likely		403 (38.3)	
**Perceptions related to COVID-19 vaccination based on the theory of planned behavior**				
	**Positive attitudes toward COVID-19 vaccination**			
		Positive Attitude Scale^a^ score, mean (SD)	8.0 (1.2)	
		COVID-19 vaccination is highly effective in protecting your child from COVID-19 (agree), n (%)	603 (57.3)	
		Taking up COVID-19 vaccination can contribute to the control of COVID-19 in China (agree), n (%)	896 (85.2)	
		China will have an adequate supply of COVID-19 vaccine (agree), n (%)	763 (72.5)	
	**Negative attitudes toward COVID-19 vaccination**			
		Negative Attitude Scale^b^ score, mean (SD)	7.7 (1.6)	
		Your child will have severe side effects after receiving COVID-19 vaccination (agree), n (%)	104 (9.9)	
		The protection of COVID-19 vaccines will only last for a short time (agree), n (%)	210 (20.0)	
		Your child is afraid of vaccination (agree), n (%)	216 (20.5)	
		You do not have time to take your child for COVID-19 vaccination (agree), n (%)	234 (22.2)	
	**Perceived subjective norm related to child’s COVID-19 vaccination: your family member would support you in having your child take up COVID-19 vaccination**			
		Response score, mean (SD)^c^	2.5 (0.6)	
		Agree, n (%)	542 (51.5)	
	**Perceived behavioral control to have the child take up COVID-19 vaccination: having the child receive COVID-19 vaccination is easy for you if you want them to**			
		Response score, mean (SD)^c^	2.3 (0.7)	
		Agree, n (%)	456 (43.3)	
**Influence of social media related to COVID-19 vaccination**				
	**Frequency of exposure to positive information related to COVID-19 vaccination (eg, new vaccines entering clinical trials, promising efficacies of the vaccines, and vaccines will enter the market soon) on social media**			
		Response score, mean (SD)^d^	2.9 (0.9)	
		Almost none, n (%)	107 (10.2)	
		Seldom, n (%)	216 (20.5)	
		Sometimes, n (%)	420 (39.9)	
		Always, n (%)	309 (29.4)	
	**Frequency of exposure to negative information related to COVID-19 vaccination (eg, concerns about efficacies and supplies, side effects of the vaccines, and receiving vaccines will cause COVID-19) on social media**			
		Response score, mean (SD)^d^	2.3 (0.9)	
		Almost none, n (%)	244 (23.2)	
		Seldom, n (%)	366 (34.8)	
		Sometimes, n (%)	327 (31.1)	
		Always, n (%)	115 (10.9)	
	**Frequency of exposure to testimonials given by participants of the COVID-19 vaccine clinical trials on social media**			
		Response score, mean (SD)^d^	1.9 (1.0)	
		Almost none, n (%)	503 (47.8)	
		Seldom, n (%)	266 (25.3)	
		Sometimes, n (%)	185 (17.6)	
		Always, n (%)	98 (9.3)	
	**Frequency of exposure to negative information about other vaccine incidents in China (eg, selling problematic vaccines and severe side effects) on social media**			
		Response score, mean (SD)^d^	2.0 (1.0)	
		Almost none, n (%)	433 (41.2)	
		Seldom, n (%)	321 (30.5)	
		Sometimes, n (%)	207 (19.7)	
		Always, n (%)	91 (8.7)	

^a^Response categories for the 3-item Positive Attitude Scale were as follows: 1 (disagree), 2 (neutral), and 3 (agree). Cronbach α=.71; one factor was identified by exploratory factor analysis, explaining 64.0% of the total variance.

^b^Response categories for the 4-item Negative Attitude Scale were as follows: 1 (disagree), 2 (neutral), and 3 (agree). Cronbach α=.64; one factor was identified by exploratory factor analysis, explaining 56.6% of the total variance.

^c^Response categories were as follows: 1 (disagree), 2 (neutral), and 3 (agree).

^d^Response categories were as follows: 1 (almost none), 2 (seldom), 3 (sometimes), and 4 (always).

### Factors Associated With Parental Acceptability of COVID-19 Vaccination

In the univariate logistic regression analysis, age of the children, self-reported avoiding of social and meal gatherings with other people who do not live together, and self-reported avoiding of crowed places were significantly associated with parental acceptability of COVID-19 vaccination (see Table 3).

**Table 3:** Associations between background characteristics and parental acceptability of free COVID-19 vaccination (N=1052).

Characteristics			Crude odds ratio (95% CI)	*P* value
**Sociodemographics**				
	**Age of the parent (years)**			
		18-30	1.0	
		31-40	1.23 (0.89-1.72)	.21
		>40	1.35 (0.90-2.03)	.14
	**Gender**			
		Male	1.0	
		Female	0.96 (0.73-1.27)	.79
	**Relationship status**			
		Married	1.0	
		Currently single or divorced	1.44 (0.68-3.05)	.34
		Having a stable boyfriend or girlfriend	1.15 (0.12-11.08)	.91
	**Highest education level attained**			
		Junior high school or below	1.0	
		Senior high school or equivalent	1.14 (0.83-1.57)	.42
		College or university or above	0.98 (0.70-1.38)	.92
	**Monthly personal income (¥)**			
		<3000	1.0	
		3000-4999	0.95 (0.67-1.34)	.76
		5000-6999	1.01 (0.66-1.56)	.95
		7000-9999	1.03 (0.60-1.79)	.91
		≥10,000	1.26 (0.68-2.32)	.46
	**Type of work**			
		Frontline worker	1.0	
		Management staff	0.92 (0.69-1.22)	.57
	**History of seasonal influenza vaccination**			
		No	1.0	
		Yes	1.35 (0.94-1.92)	.10
	**Having at least one family member with a history of COVID-19**			
		No	1.0	
		Yes	N/A^a^	N/A
	**Age of the child (years)**			
		0-3	1.0	
		4-6	1.26 (0.84-1.91)	.26
		7-12	1.41 (1.01-1.97)	.046
		13-17	1.28 (0.86-1.91)	.23
**Personal COVID-19 preventive measures in the past month**				
	**Consistent face mask wearing in public places and on transportation other than the workplace**			
		No	1.0	
		Yes	1.31 (0.93-1.86)	.13
	**Consistent face mask wearing when you have close contact with other people in workplace**			
		No	1.0	
		Yes	1.29 (0.96-1.73)	.10
	**Sanitizing hands (ie, using soaps, liquid soaps, or alcohol-based sanitizer) every time after returning from public spaces or touching public installations**			
		No	1.0	
		Yes	1.10 (0.84-1.45)	.49
	**Self-reported avoiding of social and meal gatherings with other people who do not live together**			
		No	1.0	
		Yes	1.70 (1.30-2.24)	<.001
	**Self-reported avoiding of crowed places**			
		No	1.0	
		Yes	1.58 (1.27-2.22)	<.001

^a^N/A: not applicable.

After adjusting for these significant background characteristics, positive attitudes toward COVID-19 vaccination (adjusted odds ratio [AOR] 1.70, 95% CI 1.50-1.91), perceiving that a family member would support them in having their children take up COVID-19 vaccination (AOR 4.18, 95% CI 3.21-5.43), and perceived behavioral control to have the children receive COVID-19 vaccination (AOR 1.84, 95% CI 1.49-2.26) were associated with higher parental acceptability of COVID-19 vaccination. Regarding social media influence, higher exposure to positive information related to COVID-19 vaccination was associated with higher parental acceptability of COVID-19 vaccination (AOR 1.35, 95% CI 1.17-1.56). Higher exposure to negative information related to COVID-19 vaccination was negatively associated with the dependent variable (AOR 0.85, 95% CI 0.74-0.99) (see Table 4).

**Table 4:** Factors associated with parental acceptability of a free COVID-19 vaccination (N=1052).

Factors		AOR^a^ (95% CI)	*P* value
**Perceptions related to COVID-19 vaccination based on the theory of planned behavior**			
	Positive Attitude Scale	1.70 (1.50-1.91)	<.001
	Negative Attitude Scale	0.93 (0.85-1.01)	.09
	Your family member would support you in having your child take up COVID-19 vaccination (ie, perceived subjective norm)	4.18 (3.21-5.43)	<.001
	Having your child receive the COVID-19 vaccination is easy for you if you want them to (ie, perceived behavioral control)	1.84 (1.49-2.26)	<.001
**Influence of social media related to COVID-19 vaccination**			
	Frequency of exposure to positive information related to COVID-19 vaccination on social media	1.35 (1.17-1.56)	<.001
	Frequency of exposure to negative information related to COVID-19 vaccination on social media	0.85 (0.74-0.99)	.03
	Frequency of exposure to testimonials given by participants of the COVID-19 vaccine clinical trials on social media	1.07 (0.94-1.23)	.31
	Frequency of exposure to negative information about other vaccine incidents in China on social media	0.91 (0.79-1.05)	.20

^a^AOR: adjusted odds ratio; background characteristics with *P*<.05 in the univariate analysis were adjusted in the multivariate logistic regression models.

## Discussion

This is one of the first studies investigating parental acceptability of COVID-19 vaccination in China that provides some preliminary data to inform policy making and service planning. About 70% of the Chinese parents accepted COVID-19 vaccination for their children. However, given the gap between acceptability and actual behaviors [22], effective health promotion is needed when COVID-19 vaccines become available in order to achieve high vaccine coverage among children.

Our findings provided empirical insights to inform health promotion development. More attention should be given to parents with younger children and those with lower compliance to physical distancing measures (ie, avoiding social and meal gatherings and crowed places), as they reported lower parental acceptability of COVID-19 vaccination. Parents with children attending primary or secondary schools (ie, 7-17 years of age) might have more concerns about COVID-19 transmission within schools and, hence, have higher motivation to vaccinate their children against COVID-19. Parents with higher compliance to physical distancing measures may have stronger motivation and self-efficacy to protect themselves and their children, and COVID-19 vaccination is likely to be considered a useful means for protection.

The TPB is a potentially useful framework to guide the development of future programs, as three of four TPB constructs used in this study were significantly associated with parental acceptability in expected directions. It is useful to increase positive attitudes toward COVID-19 vaccination, as this was a facilitator. In addition to the beneficial effect for their children (eg, prevent COVID-19 effectively), health communication messages should also emphasize to parents that having their children take up COVID-19 vaccination would result in herd immunization, which could contribute to COVID-19 control. Building up confidence related to vaccine supply may also be a useful strategy. Over half of participants perceived that their family members would support them in having their children take up COVID-19 vaccination. Such perception was also a facilitator. Future health promotion should enhance parents’ knowledge of COVID-19 vaccination and encourage them to discuss their children’s vaccination with other family members in order to obtain support from these significant others. It is also useful to enhance perceived behavioral control, as this was another facilitator. There is much room for improvement. Multiple strategies may be applied in future COVID-19 vaccination programs, which may include simplification of the procedures to obtain vaccination and school-based vaccination programs. Relatively few participants had concerns related to side effects, duration of vaccine protection, children’s apprehension, or other logistical issues. The associations between these concerns and parental acceptability were not statistically significant. Addressing these concerns might not be useful strategies in future promotion campaigns.

Our findings suggested that COVID-19 vaccination triggered intensive responses on social media among Chinese parents, as about 70% of the participants were sometimes or always exposed to information specific to COVID-19 vaccination on different social media platforms. Exposure to content about positive information related to COVID-19 vaccination, such as promising vaccine efficacy, was associated with higher parental acceptability. This is understandable, as this type of information can increase parents’ confidence in COVID-19 vaccines and reduce their concerns. Higher exposure to negative information about COVID-19 vaccination was associated with lower parental acceptability. Previous studies showed that people were more likely to absorb negative rather than positive information during a disease outbreak [23]. Although social media is a powerful tool for disseminating information, there are concerns related to inaccurate data, unverified rumors, and even malicious misinformation on these platforms [24]. A global epidemic of misinformation has been spreading through social media during the COVID-19 pandemic, which might pose challenges for future COVID-19 vaccination programs [25,26]. The findings highlighted the importance of transparency in communicating about the vaccine development process and vaccine safety testing. Public health authorities should also identify and verify misinformation in a timely manner. Studies showed that a major vaccine incident (ie, the Changchun Changsheng vaccine incident) had significantly impaired confidence in vaccines among Chinese people [27]. However, in our study, negative information about these vaccine incidents did not influence parental acceptability of COVID-19 vaccination.

This is one of the first studies investigating parental acceptability of COVID-19 vaccination in China. It used the TPB as a theoretical framework and the sample size was relatively large. However, it has a number of limitations. First, we only included parents who were factory workers, as this was a secondary analysis. In 2018, 34.3% of Shenzhen’s population were factory workers [28]. However, failure to include parents with other occupations or those without full-time work was one major limitation of this study and limited the representativeness of our sample. In addition, participants were recruited in one Chinese city. Generalization should be made cautiously. Second, since the study was anonymous and did not collect participants’ identification, we were not able to collect information about those who refused to join the study. Parents who refused to join the study might have different characteristics as compared to study participants. Selection bias might exist. Our response rate was relatively high as compared to other online surveys of similar topics [12]. Third, there was a lack of methodological innovation in this study. The aim of this study was to provide timely information to facilitate the promotion and service planning related to COVID-19 vaccination in China. Fourth, we did not ask about parental acceptability that was conditional on different cost scenarios. It is common for the Chinese government to provide free vaccination to priority groups in order to increase coverage. Since children were considered as one of the priority groups to receive COVID-19 vaccination, it is possible for the government to offer free vaccines to this group. Fifth, data were self-reported and verification was not feasible. Recall bias might exist. Participants might also overreport their acceptability due to social desirability. Moreover, most items and scales used in this study were self-constructed based on those from previous studies on parental acceptability of human papillomavirus vaccination in China [20]. The internal reliabilities of these scales were acceptable, but these scales may require external validation. Furthermore, this was a cross-sectional study and could not establish a causal relationship.

In sum, parents’ acceptability of COVID-19 vaccination for their children under the age of 18 years was high among Chinese parents. The TPB is a useful framework to guide the development of future campaigns promoting COVID-19 vaccination targeting parents. Enhancing positive attitudes, creating a supportive subjective norm, and increasing parents’ perceived behavioral control related to their children’s COVID-19 vaccination are potentially useful health promotion strategies. Transparency in communicating about the vaccine development process and vaccine safety testing is important. Public health authorities should also address misinformation in a timely manner.

## Abbreviations

AOR: adjusted odds ratio
CDC: Centre for Disease Control and Prevention
QR: quick response
TPB: theory of planned behavior
